# Bioartificial pulsatile cuffs fabricated from human induced pluripotent stem cell-derived cardiomyocytes using a pre-vascularization technique

**DOI:** 10.1038/s41536-022-00218-7

**Published:** 2022-03-31

**Authors:** Yuki Endo, Jun Homma, Hidekazu Sekine, Katsuhisa Matsuura, Tatsuya Shimizu, Hiroshi Niinami

**Affiliations:** 1 Department of Cardiovascular Surgery, Tokyo Women’s Medical University, 8-1 Kawada-cho, Shinjuku-ku, Tokyo, 162-8666 Japan; 2 Institute of Advanced Biomedical Engineering and Science, Tokyo Women’s Medical University, TWIns, 8-1 Kawada-cho, Shinjuku-ku, Tokyo, 162-8666 Japan

**Keywords:** Induced pluripotent stem cells, Heart failure

## Abstract

There is great interest in the development of techniques to bioengineer pulsatile myocardial tissue as a next-generation regenerative therapy for severe heart failure. However, creation of thick myocardial grafts for regenerative medicine requires the incorporation of blood vessels. In this study, we describe a new method of constructing a vascular network in vivo that allows the construction of thick human myocardial tissue from multi-layered cell sheets. A gelatin sheet pre-loaded with growth factors was transplanted onto the superficial femoral artery and vein of the rat. These structures were encapsulated together within an ethylene vinyl alcohol membrane and incubated in vivo for 3 weeks (with distal superficial femoral artery ligation after 2 weeks to promote blood flow to the vascular bed). Subsequently, six cardiomyocyte sheets were transplanted onto the vascular bed in two stages (three sheets, two times). Incubation of this construct for a further week generated vascularized human myocardial tissue with an independent circulation supplied by an artery and vein suitable for anastomosis to host vessels. Notably, laminating six cell sheets on the vascular bed in two stages rather than one allowed the creation of thicker myocardial tissue while suppressing tissue remodeling and fibrosis. Finally, the pulsatile myocardial tissue was shown to generate auxiliary pressure when wrapped around the common iliac artery of a rat. Further development of this technique might facilitate the generation of circulatory assist devices for patients with heart failure.

## Introduction

Heart failure can arise from various causes including coronary artery disease, hypertension, dilated cardiomyopathy, hypertrophic cardiomyopathy, and restrictive cardiomyopathy^1^. The most effective treatment for end-stage heart failure is cardiac transplantation^2^, but the number of transplants that can be performed each year is limited by a shortage of donors^3^. The development of implantable ventricular assist devices has provided an alternative treatment option for patients with severe heart failure, but the mechanical devices used currently are associated with various adverse effects that can lead to poor outcomes in some patients^4^.

Progress in the field of regenerative medicine has raised hopes that bioengineered tissues might be viable alternatives to donor hearts in the future^5^. Cardiac-like tissue with limited functionality has been generated by seeding cardiomyocytes on pre-formed scaffolds such as artificial polymers or decellularized tissues^6,7^, while functional myocardial tissue has been created using hydrogel-based approaches^8,9^. We have been developing a scaffold-free method that utilizes cell sheets to generate heart tissue with a high cellularity^10,11^. Our technique involves the culture of cardiomyocytes on a temperature-responsive intelligent surface, which allows an intact cell sheet to be harvested from the culture dish simply by reducing the temperature. An important advantage of our approach is that it avoids the use of proteolytic enzymes and thus retains the cell adhesion proteins. Furthermore, cell sheets can be stacked to produce a three-dimensional (3D) construct and directly transplanted onto host tissue in vivo without the need for suturing. We have successfully generated cardiac cell sheets from human induced pluripotent stem cells (hiPSCs)^12^. Triple-layered constructs (each made by stacking three hiPSC-derived cell sheets) were able to survive and beat spontaneously after implantation onto the subcutaneous tissues of rats^13^, and multi-step transplantation at intervals enabled the fabrication of tissue with a thickness of 1 mm^14^. Furthermore, cell sheets were shown to form gap junctions with the host heart after in vivo transplantation into the rat^15^ and improve cardiac function in a rat model of myocardial infarction^16^. However, it is currently believed that the benefits of a several-layer cell sheet arise mainly from paracrine effects due to the secretion of growth factors by the transplanted cells. The manufacture of cardiac grafts that directly contribute contractile force to help pump blood around the body will necessitate the development of techniques to generate much thicker tissues.

Bioengineered myocardial constructs that rely on diffusion for the supply of oxygen are limited to a thickness of only 100–200 μm. Since a multi-step transplantation process would be impractical for clinical use in patients, there has been great interest in the development of techniques to generate thick myocardial tissue with a perfusable vascular bed. Previously, we have reported the use of cell sheet technology and an in vitro bioreactor system to create cardiac tissue with a perfusable vascular bed derived from rat femoral muscle^17^ or porcine small intestine^18^. Here, we describe an in vivo pre-vascularization method that can be used to construct a thin vascular bed with an abundant capillary network. Stepwise stacking of cell sheets on this vascular bed allowed us to generate cardiac tissue with a total thickness (myocardial component and vascular bed) of ~1.5 mm. Furthermore, we were able to create a ‘cuff’ of human myocardial tissue from multi-layered cell sheets that could function as an arterial pump. We anticipate that further development of our technique could allow the bioengineering of circulatory assist devices for use in the treatment of heart failure.

## Results

### Preparation of a vascular bed for myocardial tissue construction in an in vivo environment

**Fig. 1:**
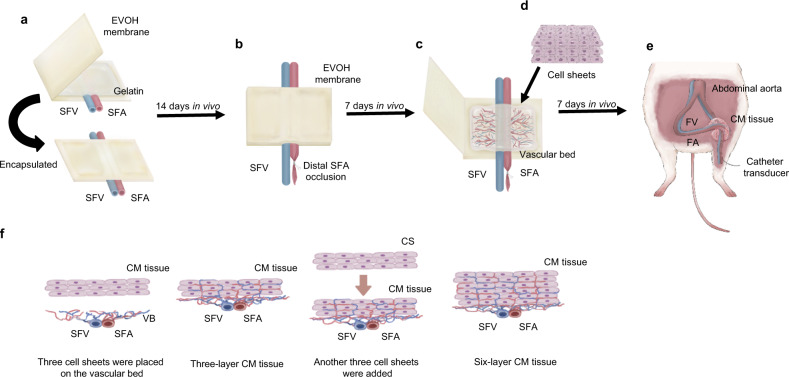
Construction of the vascular bed and cardiomyocyte tissue. **a** A skin incision was made in the inguinal region, and the SFA and SFV were dissected from the surrounding tissue to create a pedicle. Gelatin gel containing growth factors was placed on the blood vessels and encapsulated in an EVOH membrane. **b** The distal side of the SFA was ligated after the gelatin gel had been incubated in the rat for 14 days. **c** The EVOH membrane was opened after incubation for a further 7 days to confirm the presence of new blood vessels in the gelatin gel (i.e., the formation of a vascular bed). **d** Three hiPSC-derived cardiomyocyte sheets (constructed in vitro) were transplanted onto the vascular bed, encapsulated in an EVOH membrane and incubated in the rat for 24 h. Then, the EVAL membrane was opened, and an additional three cardiomyocyte sheets were transplanted onto the original cell sheets. The vascular bed and six layers of cell sheets were encapsulated in an EVOH membrane and incubated in vivo for 7 days. **e** Seven days after transplantation, the cardiomyocyte (CM) tissue was mobilized and wrapped around the contralateral CIA in the same rat. Pressure generated in the CIA by contraction of the graft tissue was measured with a catheter transducer. **f** Schematic illustration of the two-step procedure used to generate cardiomyocyte (CM) tissue from six cell sheets. Three cell sheets were stacked, and the triple-layered construct was placed on the vascular bed (VB) and perfused in vivo for 24 h. Then, another triple-layered cell sheet was laid onto the first graft, and the construct was perfused for a further 7 days to allow it to become thoroughly vascularized.

The construction of thick and functional human myocardial tissue in vivo requires that the cardiomyocyte sheets obtain blood flow from the host soon after transplantation to ensure their survival. We considered it important for the cell sheet-derived tissue to have a vascular bed that would allow it to be integrated into the systemic vasculature. To achieve this aim, we prepared a vascular bed with a surgically anastomosable artery and vein (Fig. 1). The vascular bed was constructed using thin gelatin sheets that slowly released vascular growth factors to promote the proliferation of new blood vessels and the generation of capillaries within the gelatin gel. The gelatin was encapsulated in a non-adhesive membrane to ensure that the vascular bed would not adhere to host tissue in vivo. Then, a triple-layered cardiomyocyte sheet was transplanted onto the vascular bed. After an interval to allow blood vessels to extend into the transplanted graft, a second three-layer cardiomyocyte sheet was transplanted onto the first to construct a thick portion of human cardiomyocyte tissue. Finally, we investigated whether this construct could be wrapped around an artery as a pulsatile cuff that functioned as a pump.

**Fig. 2:**
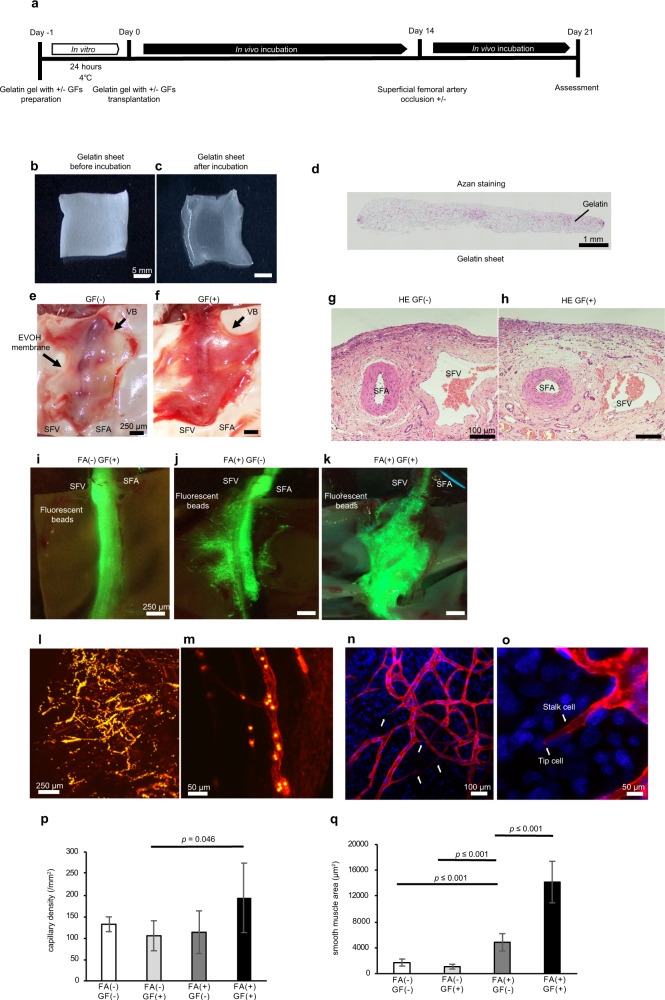
Evaluations of vascular beds produced under various experimental conditions. **a** Schematic overview of the protocol used to construct the vascular bed. GFs, growth factors. **b** A gelatin sheet before incubation with growth factors. **c** The gelatin sheet after incubation with growth factors at 4 °C for 24 h. **d** Azan staining of the gelatin sheet before incubation. **e** The vascular bed (VB) in the GF(−) group (gelatin sheet not pre-loaded with growth factors) 21 days after incubation in vivo. **f** The vascular bed (VB) in the GF(+) group (gelatin sheet pre-loaded with growth factors) 21 days after incubation in vivo. **g** Section of the vascular bed (VB) in the GF(−) group stained with hematoxylin and eosin (HE) on day 21. **h** Section of the vascular bed (VB) in the GF(+) group stained with HE on day 21. The region around the SFA and SFV contained new blood vessels rich in smooth muscle. **i, k** Fluorescent beads (4 μm diameter) were administered into the SFA and vascular bed via a catheter inserted through the abdominal aorta and advanced into the femoral artery (day 21). New blood vessels in the gelatin gel were more extensive in the FA(+) GF(+) group (gelatin sheet pre-loaded with growth factors, SFA occluded after 14 days of incubation in vivo) than in the FA(−) GF(+) group or FA(+) GF(−) group. **l**, **m** Tomato lectin (to label vascular endothelial cells) and fluorescent beads (to reveal blood flow) were administered to a vascular bed from the FA(+) GF(+) group and observed using two-photon microscopy. Endothelial cells in the gelatin sheet formed a network of vascular structures through which fluorescent beads passed. **n**, **o** Tomato lectin (to label vascular endothelial cells) and Hoechst solution (to label nuclei) were administered to a vascular bed from the FA(+) GF(+) group and observed using two-photon microscopy. The new blood vessels contained structures resembling tip cells and stalk cells. Stained nuclei were observed at the tips of the extending blood vessels. **p** Capillary density in the vascular bed (mean ± SEM). **q** Smooth muscle cell area in the vascular bed (mean ± SEM).

To create a vascular bed with a feeding artery and draining vein, the superficial femoral artery (SFA) and superficial femoral vein (SFV) of a rat were carefully dissected to free them from the surrounding tissues, and a prepared gelatin sheet was transplanted over the blood vessels. The gelatin sheet, which decomposes within 4 days in vivo, had a thickness of about 1 mm and provided the sustained release of growth factors to promote the genesis of capillaries. The gelatin gel and adjacent portions of the blood vessels were encapsulated in a porous ethylene vinyl alcohol (EVOH) membrane that prevented adhesion of the vascular bed to the surrounding tissues. In vivo incubation of the vascular bed was initially performed for 2 weeks (Fig. 1a). Then, the encapsulated SFA distal to the gelatin gel was ligated with a suture (Fig. 1b), and in vivo incubation was continued for a further week to increase the blood flow to the new capillaries in the gelatin sheet and promote the maturation of blood vessels in the vascular bed (Fig. 2a).

### Comparisons of vascular beds generated under different conditions

We first carried out experiments to establish whether the inclusion of growth factors within the gelatin sheet (*n* = 6) and ligation of the SFA after 7 days of incubation (*n* = 5) would enhance the development of blood vessels in the vascular bed. The gelatin gel (Fig. 2b–d) was incubated either with vascular endothelial growth factor (VEGF) and fibroblast growth factor-2 (FGF-2) or with phosphate-buffered saline (PBS, used as the control) at 4 °C for 24 h. Macroscopic and histological observations of the EVOH membrane 21 days after transplantation in vivo revealed that the addition of growth factors to the gelatin sheet increased the blood flow in the vascular bed (Fig. 2e, f) as well as the number of capillaries and the degree of vascular maturity (Fig. 2g, h). The administration of fluorescent beads (via a catheter) into the femoral artery revealed that SFA ligation promoted the formation of new blood vessels in the gelatin gel that were connected to the systemic circulation, and the network of new vessels was more extensive when the gelatin sheet was pre-loaded with growth factors (Fig. 2i–k, Supplementary Movie 1). When growth factor loaded gelatin sheets and SFA ligation were used to prepare the vascular bed, the administration of tomato lectin (to label vascular endothelial cells) and fluorescent beads (to reveal blood flow) showed that the gelatin sheet contained a network of endothelial cells that formed interconnecting vessel-like structures through which fluorescent beads could pass (Fig. 2l, m).

Quantitative evaluations revealed that the capillary density of the vascular bed was 132 ± 17/mm^2^ in the FA(−) GF(−) group (i.e., SFA not ligated and growth factors not included), 104 ± 34/mm^2^ in the FA(−) GF(+) group, 113 ± 50/mm^2^ in the FA(+) GF(−) group, and 193 ± 81/mm^2^ in the FA(+) GF(+) group (Fig. 2p). Notably, capillary density was significantly higher in the FA(+) GF(+) group than in the FA(−) GF(+) group (*p* = 0.046). We also assessed blood vessel maturity by calculating the area of vascular smooth muscle cells identified by immunohistochemistry using an anti-α-smooth muscle actin (SMA) antibody (Fig. 2q). Smooth muscle area in the FA(+) GF(−) group (4680 ± 560 μm^2^) was significantly larger than that in the FA(−) GF(−) group (1715 ± 547 μm^2^, *p* = 0.001) and FA(−) GF(+) group (1079 ± 367 μm^2^, *p* = 0.0005). Furthermore, the FA(+) GF(+) group had a substantially larger smooth muscle area than the other three groups (14200 ± 1360 μm^2^; *p* < 0.001). Based on the above data, we concluded that the quality of the vascular bed was improved by loading the gelatin sheet with growth factors and ligating the distal SFA after 14 days.

### Stacking triple-layered cardiomyocyte sheets on the vascular bed in a two-stage process produces 3D myocardial tissue with blood vessels

**Fig. 3:**
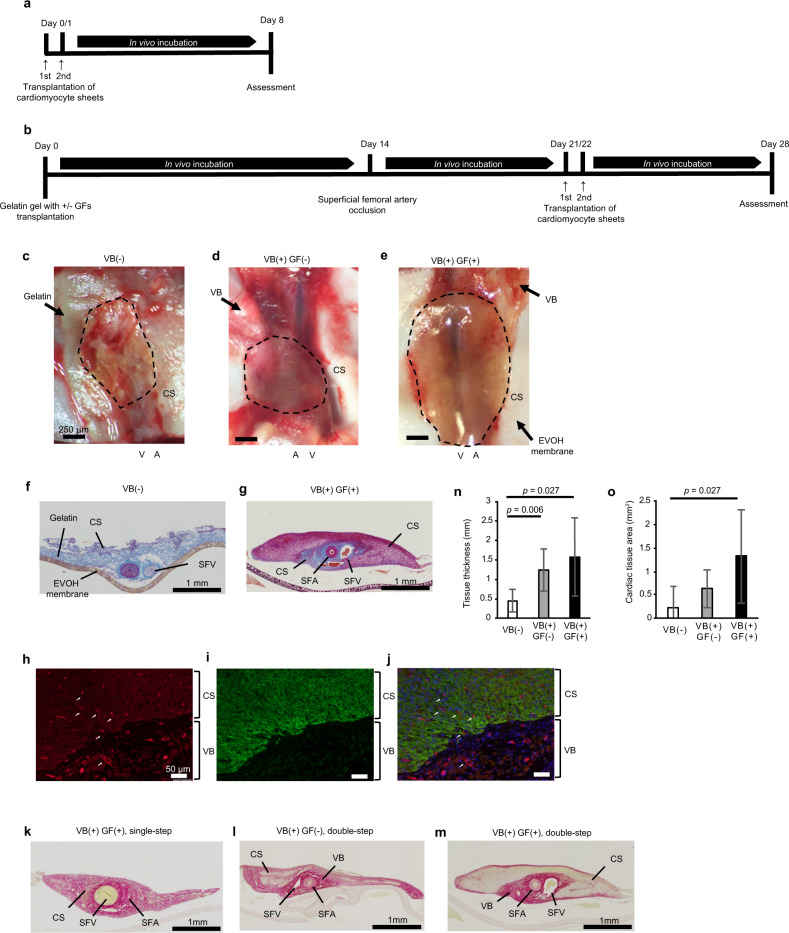
Comparisons of myocardial tissues bioengineered under various conditions. **a** Two-step protocol used to generate human myocardial tissue in vivo without a vascular bed. Three-layer cardiomyocyte sheets were transplanted on days 0 and 1. **b** Two-step protocol used to generate human myocardial tissue on a vascular bed (preincubated for 21 days with SFA ligation on day 14). Three-layer cardiomyocyte sheets were transplanted on days 21 and 22. GFs, growth factors. **c, e** Photographs obtained 7 days after the two-step transplantation of six cardiomyocyte sheets (CS) in vivo. VB(−) group (without prior induction of a vascular bed): the transplanted gelatin gel retained its original shape, but the myocardial sheets had not engrafted (**c**). VB(+) GF(−) group (prior induction of a vascular bed without growth factors): myocardial sheet engraftment was not evident (**d**). VB(+) GF(+) group (prior induction of a vascular bed with growth factors): myocardial tissue had engrafted onto the vascular bed and contained capillaries (**e**). A artery; V vein; VB vascular bed. **f**, **g** Tissue sections stained with Azan. VB(−) group: gelatin (blue) containing the SFA and SFV was observed on the EVOH membrane, but myocardial tissue was not observed (**f**). VB(+) GF(+) group: the cardiomyocyte sheets had engrafted onto the vascular bed (blue) (**g**). **h, j** Tissue sections immunostained for RECA1 (red) and cTnT (green). Nuclei were counterstained with DAPI (blue). The merged image (**j**) shows new blood vessels extending from the rat vascular bed into the human myocardial tissue. **k, m** Sections stained with Sirius red to detect connective tissue (i.e., fibrosis). Myocardial tissue fibrosis was observed following one-stage transplantation of six cardiomyocyte sheets onto a vascular bed containing growth factors (**k**) or two-step transplantation of six cardiomyocyte sheets onto a vascular bed made without growth factors (**l**). Myocardial tissue fibrosis was not evident following two-stage transplantation of six cell sheets onto a vascular bed containing growth factors, although connective tissue was detected on the graft surface (**m**). **n** Transplanted tissue thickness (mean + SEM). VB(−), *n* = 5; VB(+) GF(−), *n* = 8; VB(+) GF(+), *n* = 7. **o** Myocardial tissue area (mean ± SEM).

Our previous study reported that thick, 3D tissue could be generated in vivo by repeatedly transplanting three-layer cell sheets (i.e., three cell sheets stacked on each other) at 1-day intervals onto rat subcutaneous tissue^14^. Therefore, we examined whether a similar approach could be used to generate myocardial tissue on the gelatin sheet-based vascular bed. In our initial experiments, a three-layer cardiomyocyte sheet was transplanted in vivo under three different conditions: (1) onto a gelatin sheet that had not been preincubated for 21 days [VB(−) group]; (2) onto a growth factor-free gelatin sheet that had been preincubated for 21 days to produce a vascular bed [VB(+) GF(−) group]; and (3) onto a growth factor-loaded gelatin sheet that had been preincubated for 21 days to generate a vascular bed [VB(+) GF(+) group]. A two-step protocol for cell sheet transplantation was used in each case: a three-layer cardiomyocyte sheet was transplanted first, another three-layer cardiomyocyte sheet was transplanted 24 h later, and the construct was then incubated in vivo for a further 7 days (Fig. 3a, b).

Pulsatile tissue was not observed in the VB(−) group, and there was little or no evidence of myocardial sheet engraftment (Fig. 3c). Although pulsatile tissue was observed in the VB(+) GF(−) group, engraftment of the myocardial cell sheets could not be confirmed (Fig. 3d). However, the myocardial tissue in the VB(+) GF(+) group was pulsatile, had successfully engrafted onto the vascular bed and contained capillaries (Fig. 3e). The myocardial tissue graft could not be identified in tissue sections stained with Azan in the VB(−) group (Fig. 3f), whereas engraftment of the myocardial sheets was evident in the VB(+) GF(+) group (Fig. 3g). Furthermore, double-immunostaining for cardiac troponin T (cTnT) and rat endothelial cell antigen-1 (RECA1) confirmed that new blood vessels had migrated from the vascular bed into the human myocardial tissue (Fig. 3h–j).

Insufficient delivery of oxygen and nutrients to a graft will result in fibrosis. Therefore, we evaluated whether the transplanted cardiomyocyte sheets had undergone fibrosis by staining for connective tissue with Sirius red. We observed obvious staining of the myocardial tissue with Sirius red when six cardiomyocyte sheets were transplanted onto the vascular bed in one step in the presence of growth factors (Fig. 3k) or in two steps in the absence of growth factors (Fig. 3l), indicating that fibrosis had developed in both cases. However, little or no fibrosis was detected within the myocardial tissue when six cell sheets were transplanted in two stages onto a vascular bed containing growth factors, although some connective tissue was evident on the graft surface (Fig. 3m).

We also made quantitative assessments of tissue thickness and cardiac tissue area to further evaluate transplantation success. Total tissue thickness (Fig. 3n) was significantly greater in the VB(+) GF(+) group (1.57 ± 1.01 mm) and VB(+) GF(−) group (1.24 ± 0.54 mm) than in the VB(−) group (0.45 ± 0.29 mm; *p* = 0.027 and *p* = 0.006, respectively). The thickness of the myocardial component was 423.5 ± 172.9 μm in the VB(+) GF(+) group but non-evaluable in the VB(−) group because little residual myocardium was found. Cardiac tissue area (Fig. 3o) was also significantly larger in the VB(+) GF(+) group than in the VB(−) group (1.32 ± 1.00 mm^2^ vs. 0.21 ± 0.36 mm^2^; *p* = 0.027). When considered together, the results described above demonstrate that thick, vascularized human myocardial tissue can be generated in vivo by the stepwise stacking of cardiomyocyte cell sheets on a vascular bed prepared with growth factors.

### Potential utility of the bioengineered human myocardial tissue as a circulatory assist device

**Fig. 4:**
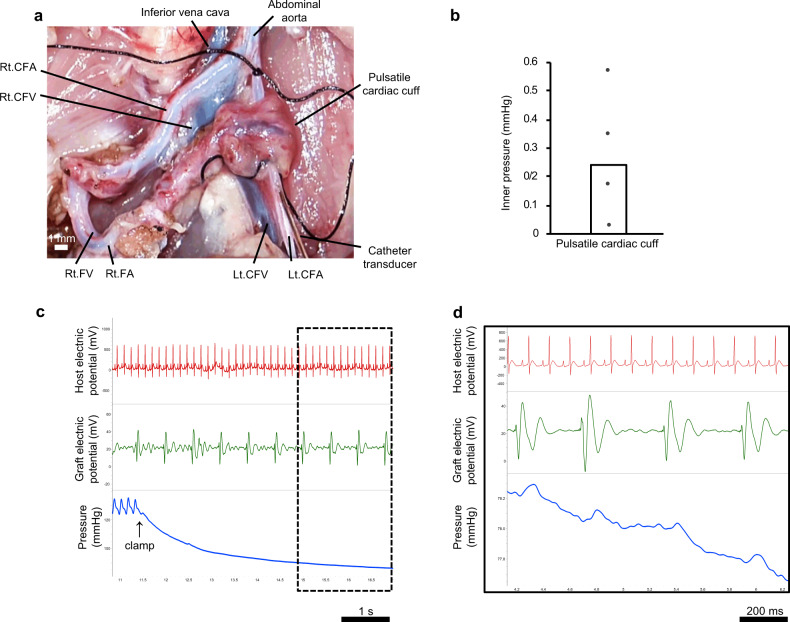
Evaluation of the myocardial tissue as an auxiliary pump. **a** Myocardial tissue prepared in vivo was mobilized and wrapped around the contralateral CIA. A catheter with a pressure transducer was inserted from the distal side of the CIA, and the internal pressure generated by the pulsation of the myocardial tissue was measured. **b** Pressure generated within the rat CIA by contraction of the human myocardial tissue cuff. Each point represents the pressure measured in an independent experiment, and the bar shows the mean value for the four experiments. **c**–**d** Representative traces showing the host electrocardiogram (upper), electric potentials generated by the graft (middle), and pressure in the CIA (lower).

Finally, we evaluated whether human myocardial tissue generated on a growth factor-supplemented vascular bed with the two-step transplantation method might have functionality as a circulatory assist device. The myocardial tissue was mobilized with its blood supply intact (see Methods for details) and wrapped tightly as a cuff around the contralateral common iliac artery (CIA), which had been carefully dissected away from the surrounding tissue (Fig. 1e). A catheter transducer was inserted from the distal side of the CIA to measure the pressure generated within the CIA by the myocardial tissue cuff, and the abdominal aorta was clamped to create a closed circuit (Fig. 4a, b and Supplementary Movie 2). The electric potentials of the graft tissue and the host electrocardiogram were also recorded (Fig. 4c). The pressure generated by each contraction of the graft was 0.24 ± 0.20 mmHg (*n* = 4; Fig. 4d). This result is proof of concept that the vascularized human myocardial tissue produced by our technique has the flexibility to be wrapped around an artery and the functionality to act as an auxiliary circulatory pump.

## Discussion

The present study describes the use of cell sheet engineering and a new pre-vascularization method to generate functional vascularized human myocardial tissue with a feeding artery and draining vein.

We utilized an in vivo incubation method to construct a vascular bed with an abundant and independent vascular network. Since the aim of this research was to create a vascularized thick graft that could be transplanted to another site, it was vital that the thin and flexible vascular bed was prevented from adhering to the surrounding tissues. Previous studies investigating the encapsulation of cells and tissues utilized an oxygen-permeable silicone membrane^19^ and cytokine-administered soft tissues^20^, but these approaches were associated with excessive growth of collagen in and around the tissue. Therefore, we used a porous EVOH membrane to isolate the vascular bed from the surrounding tissues. EVOH membranes have excellent biocompatibility because they are hydrophilic, uncharged, retain structural water, and have a smooth surface with few non-specific protein bonds. In addition, EVOH membranes exert little or no effects on the coagulation and thrombolytic cascades^21^. Silicone membranes were not used in this study because they are considered more bioinvasive than EVOH membranes and induce a strong inflammatory reaction that produces excess collagen. Indeed, we found that the reaction layer formed on human cardiomyocyte sheets was significantly thinner when an EVOH membrane was used than when a 500 μm-thick silicone membrane was utilized (Supplementary Fig. 1a, b). Furthermore, an EVOH membrane can protect encapsulated cells because it is very thin (90–100 μm; Fig. 3f, g), has excellent biocompatibility and allows selective permeation^22^. Therefore, we speculated that an EVOH membrane would help the survival of transplanted cardiomyocytes. However, when a six-layer cardiomyocyte sheet (with a thickness of about 500 μm) was transplanted onto a non-prevascularized bed (i.e., onto a gelatin sheet placed over the SFA and SFV but not preincubated in vivo for 3 weeks), the cells died despite their encapsulation in an EVOH membrane (Fig. 3f). Cell death likely occurred because the diffusion of oxygen and nutrients through the EVOH membrane was insufficient to meet the needs of the cardiomyocytes, which have a high oxygen demand. On the other hand, myocardial tissue with a thickness of about 1.5 mm was successfully realized when the cardiomyocyte sheets were encapsulated on a vascular bed containing a rich and independent capillary network (which was fabricated by placing a growth factor-loaded gelatin gel on the superficial femoral vessels and incubating for 3 weeks in vivo). The high biocompatibility of an EVOH membrane makes it ideally suited for use as a barrier to prevent the adhesion of a vascularized graft to the surrounding tissues during incubation in vivo.

Femoral artery ligation and the inclusion of growth factors in the gelatin sheet were both found to increase the amount of vascular smooth muscle, indicating that some of the new vessels formed in the vascular bed had matured into arterioles and venules. VEGF and bFGF are vascular growth factors that stimulate angiogenesis^23–26^. Ligation of the SFA during incubation blocks the original blood flow path and forces blood into the new vessels. We speculate that this diversion of blood flow caused mechanical stimulation of endothelial cells in the immature blood vessels, which led to the activation of intracellular signaling cascades that promoted vascular maturation^27,28^.

As for the origin of neovascularization to myocardial tissue graft, immunostaining demonstrated positive staining for RECA1 in the vascular bed and myocardial tissue (Fig. 3h–j), which makes it likely that the vascular endothelial cells in the myocardial tissue had originated from the vascular bed that was connected to the host femoral artery and vein. Since vascular smooth muscle cells exhibit enhanced proliferative and migratory ability when blood vessels thicken in response to an increased blood demand such as that which occurs under ischemic conditions^24,25^ we believe that the smooth muscle cells likely migrated into the tissue after angiogenesis. The hiPSC-derived cells used in the present study were predominantly cardiomyocytes. Since the cells expressed the puromycin resistance gene under the control of the α-MHC promoter, purification of the cardiomyocytes was achieved by treatment with puromycin after cardiac differentiation. The cells obtained using this purification technique are mainly cardiomyocytes (91.2 ± 8.3%) but also include a small proportion of fibroblasts and CD31-positive endothelial cells^29^. Thus, the cell sheets utilized in the present study contained only a small number of hiPSC-derived endothelial cells.

Ischemic cell death causes necrosis and inflammatory changes due to cell membrane destruction and the release of intracellular constituents. Pattern recognition receptors (PRRs) elicit an inflammatory response when they detect damage-associated molecular patterns (DAMPs) derived from dying cells^30^. DAMPs are recognized through PRRs such as Toll-like receptors and receptor for advanced glycation end products (RAGE), and these PRRs induce various inflammatory cytokines through nuclear factor-κB and inflammasome activity. Severe tissue damage and chronic production of inflammatory cytokines prolong the interactions between parenchymal and stromal cells, leading to loss of the normal repair mechanism, fibrosis and irreversible organ dysfunction. Previously, we found that ischemic necrosis did not occur in the center of a bioengineered tissue when cell sheets were stacked at 24 h intervals and capillaries were induced to introduce a vascular network^14^. In the present study, simultaneous transplantation of six cardiomyocyte sheets generated myocardial tissue that exhibited marked fibrosis throughout, whereas little fibrosis occurred when the cell sheets were transplanted in two stages. It is likely that the one-step transplantation method failed because the thickness of the six cell sheets (about 200 μm) exceeded the limit for sufficient diffusion of oxygen and nutrients, which resulted in ischemia, cardiomyocyte death, inflammation and fibrosis. On the other hand, the two-step procedure allowed sufficient time for the first three cardiomyocyte sheets to acquire a vascular network from the vascular bed before the next three sheets were transplanted, thereby preventing severe ischemia (Supplementary Fig. 1c–e). We have evaluated the degree of tissue inflammation 3 days after cardiomyocyte sheet transplantation by immunostaining for the NF-κB subunit, p65. NF-κB is a transcription factor that regulates the expression of various inflammatory cytokines and inflammation-related molecules. Activation of NF-κB occurs following phosphorylation-induced, proteasome-mediated degradation of IκB, which allows NF-κB to translocate from the cytoplasm to the nucleus^31,32^. We performed our analysis 3 days after myocardial sheet transplantation (before the occurrence of fibrosis) because the acute inflammatory response usually peaks within the first 24 h and ends after 7 days^33^. We found that the use of a vascular bed reduced the number of myocardial tissue cells expressing p65 in their nuclei (Supplementary Fig. 1f, g), which would be consistent with a lower level of inflammation.

In this study, triple-layered cell sheets were transplanted at 1-day intervals onto a vascular bed that had been prepared over 21 days. As shown in Fig. 2k, the formation of blood vessels within the entire gelatin layer required 21 days; this long period of time was needed to allow new capillaries to develop from the SFA, which is a large, unbranched vessel. On the other hand, only a short period of time (1 day) was required for the induction of blood vessels into the cell sheet, since this angiogenesis occurred from the capillaries that had developed in the vascular bed. The difference in the time required for angiogenesis to occur in the gelatin layer (21 days) and cell sheet (1 day) was related to whether a capillary network was present just below the surface: the cell sheets were transplanted onto tissue that already contained a capillary network (pre-formed in the vascular bed), whereas this was not the case for the gelatin sheet. Previous research has confirmed that cell sheets contain new capillaries 24 h after transplantation, which allows the creation of thick tissues through the stepwise stacking of multiple cell sheets^13^. In the present study, staining with Sirius red on day 3 (Supplementary Fig. 1e) and day 7 (Fig. 3m) after cardiac sheet transplantation did not reveal the presence of fibrosis within the cardiomyocyte tissue, indicating that a transplantation interval of 1 day prevented the formation of a reaction membrane (collagen layer) between the transplanted cell sheets. Therefore, a time interval of 1 day between the transplantation of triple-layered cell sheets was sufficient to prevent ischemia.

One overarching aim of our research is to bioengineer human myocardial tissue that can be transplanted around a major blood vessel to act as an auxiliary pump. Previously, we have created pressure-generating myocardial tubes by sequentially wrapping neonatal rat cardiomyocyte sheets around the aorta of the rat^34^. Furthermore, hiPSC-derived cardiomyocyte sheets wrapped in triplicate around the inferior vena cava of the rat were shown to generate an internal pressure of 9–12 mmHg at 1 month after implantation due to maturation of the cardiomyocytes over time^29^. In the present study, we tested whether the vascularized myocardial tissue graft could be mobilized with its blood supply intact and wrapped around a major artery (the CIA) to act as an auxiliary pump. We confirmed that the myocardial tissue cuff was able to contract spontaneously and generate pressure within the CIA. Since transplanted myocardial tissue matures over time, it would be expected that the pressure generated by the myocardial tissue cuff would increase progressively. Moreover, the pressure produced by the graft could be further enhanced by increasing the number of cardiac cell sheets used in its construction. In addition, cardiomyocytes derived from hiPSCs are considered to have characteristics of pacemaker cells because they exhibit automatic activity. If the cells were not coupled directly to the heart, an artificial pacemaker could be used to drive the bioengineered graft at an appropriate beating rate that matched that of the host, irrespective of whether or not the cells in the graft had matured sufficiently to stop beating spontaneously.

The present research provides proof of concept that a myocardial tissue graft constructed with our technique can function as an auxiliary pump. One important advantage of our construct is that its feeding artery and draining vein could be anastomosed to other vessels (such as the internal thoracic arteries and veins) to supply it with blood after transplantation. Furthermore, the portability of the graft makes it well suited for use in situations where surgical access to the target vessel (e.g., pulmonary artery) is difficult and working space is limited. In addition, the force generated by such a graft would likely increase over time as the transplanted tissue matured in vivo^29^, potentially providing a long-term treatment for heart failure.

This study has some limitations. First, the internal pressure generated by the myocardial tissue cuff was too small to offer clinically relevant hemodynamic support. However, it should be noted that the graft was not fully mature and created from only six cardiomyocyte sheets. We believe that it will be relatively straightforward to increase the pumping ability of the graft by stacking a greater number of cell sheets in a stepwise manner and allowing the graft to mature over time. Second, our method was surgically invasive because it necessitated multiple operations in the inguinal region to create the vascularized myocardial tissue graft. However, since our protocol is capable of constructing vascularized tissue on a flat surface, it could be used to produce grafts on tissues that are more accessible than the inguinal vessels such as the latissimus dorsi muscle^35^, which would reduce the complexity of the procedure. In addition, since the latissimus dorsi muscle is used clinically as a free flap for reconstruction surgery, occlusion of its distal artery during graft construction would likely have no major effects on the perfusion of other important tissues, which is not the case for occlusion of the SFA. Furthermore, an important aim of our ongoing research is to construct a vascularized graft in vitro using a tissue perfusion bioreactor^17^. In the future, we envisage that selection of an alternative in vivo site and advances in in vitro techniques will reduce the surgical complexity of graft construction. Third, the inclusion of GF in the gelatin sheet was not sufficient for graft construction in the absence of SFA ligation (Fig. 3n, o). However, the quantity of vascular smooth muscle was significantly higher in the FA(+) GF(+) group than in the FA(+) GF(−) group (Fig. 2q), indicating that the administration of GF contributed to the maturation of the newly formed blood vessels. Although GF alone was not sufficient for myocardial cell sheet engraftment in this study, it is possible that adjustment of GF amounts and/or types might further enhance vascular bed maturation and potentially enable the construction of myocardial tissue grafts without the need for distal artery occlusion. Fourth, present study, we did not evaluate the characteristic findings of sarcomere structure and maturation of myocardial tissue grafts. However, previous studies have clarified the structure of myocardial tissue after transplantation of hiPSC-derived cardiomyocyte sheets into rat subcutaneous vascular bed^13^. The study showed that 2 weeks after in vivo transplantation, human myocardial tissue prepared from hiPSC-derived cardiomyocyte sheets contained cells with a striated appearance indicating microvessels and sarcomeres. Electron microscopy also showed that the myofibrils became denser, wider, and more elongated over time. At 6 months after transplantation, desmosomes had formed and the number of mitochondria had increased. Further insights into myocardial tissue maturation in vivo showed that mRNAs for various proteins involved in contraction (MYL2, MYH6, MYL7, TNNT2, RYR2) were expressed in myocardial tissue at 4 weeks after transplantation^29^. Furthermore, the mRNA expressions of MYL2, MYH6, MYL7, MYH7, TNNT2 and RYR2 in the cardiac tissue were remarkably upregulated at 8 weeks after transplantation when compared with 4 weeks after transplantation. These findings were interpreted as showing maturation of the cardiac tissue over time after transplantation in vivo. Although the present study did not examine the phenotypic properties of hiPSC-derived cardiomyocytes on the vascular bed, we would expect the myocardial cells to exhibit similar structural features and maturation characteristics to those reported by the previous investigations described above.

In conclusion, we have used cell sheet-based tissue engineering to construct a thick, flexible and vascularized graft comprising hiPSC-derived myocardial tissue. Importantly, the graft would be suitable for transplantation in vivo because it has a feeding artery and draining vein that could be anastomosed with host blood vessels. Moreover, the graft was shown to function as an auxiliary pump when placed as a cuff around a major artery. Further improvements to this technique to increase graft size and allow graft construction in vitro may enable the development of new circulatory assist devices for patients with heart failure. In addition, the vascular bed could be used to generate other tissues with a high oxygen demand such as those of the liver and kidney.

## Methods

All animal experiments were performed in accordance with the “Guidelines of Tokyo Women’s Medical University on Animal Use” and were approved by the Ethics Committee for Animal Experimentation of Tokyo Women’s Medical University. All animals were maintained on a 12 h day/night cycle with free access to food and water.

### Construction of a vascular bed

The vascular bed was created using Male Jcl:SD rats weighing 270–470 g (Clea Japan). A bioabsorbable gelatin hydrogel (Nitta Gelatin) was cut into 12 mm × 12 mm pieces and incubated in 200 mL PBS containing/not containing cytokines (1.0 μg bFGF and 0.01 μg VEGF; Nacalai Tesque) at 4 °C for 24 h. The Jcl:SD rat was anesthetized by inhalation of 2% isoflurane, and the SFA and SFV were partially isolated from the surrounding tissue. A porous EVOH membrane (supplied by Kuraray) was placed under the SFA and SFV, and the gelatin gel (prepared the day before) was transplanted onto the SFA and SFV. The EVOH membrane was folded over the top of the gelatin sheet, and the edges of the EVOH membrane were sealed with heat to encapsulate the gelatin sheet and underlying blood vessels. Two weeks later, the SFA was ligated distal to the gelatin sheet using 6-0 silk (Ethicon). The vascular bed created in the gel was evaluated after an additional week of incubation, and the rat was then euthanized with an overdose of pentobarbital.

### Assessment of flow in the vascular bed

The abdomen of the rat was opened on day 21, and all vessel branches from the abdominal aorta down to the SFA were heat coagulated or ligated with 6-0 silk. Heparin (500 units) was administered by subcutaneous injection, and a catheter (Braintree Scientific) was inserted through the abdominal aorta. The tip of the catheter was advanced under direct vision until it reached the vascular bed, and the catheter was secured within the vessel using 6-0 silk, with care taken not to occlude the catheter. Tomato lectin (DyLight 594-conjugated Lycopersion esculentum Lectin; Funakoshi) and fluorescent beads (FluoSpheres^TM^ sulfate, 4.0 μm, yellow-green; Life Technologies) were administered via the catheter (1 mL of each, diluted tenfold), and the proximal SFA and SFV were ligated with 6-0 silk to prevent the loss of the injected markers from the vascular bed. In other experiments, the same technique was used to administer Hoechst 33342 solution (Dojindo Laboratories) and tomato lectin (1 mL of each, diluted 10-fold). The vascular bed was resected, fixed with formalin (Muto Pure Chemicals) and observed using a two-photon laser-scanning microscopy system (FV1000MPE, Olympus) equipped with a 25× objective lens (XLPLN25XWMP, numerical aperture = 1.05, working distance = 2.0 mm) and Fluoview 3.1 software. The brightness compensation function in the *z*-direction was used to change the detector sensitivity and laser power. A series of x–y images were obtained to allow 3D reconstruction of the data (volume dimensions: 500 μm × 500 μm × 200 μm).

### Measurement of capillary density and smooth muscle cell area in the vascular bed

The vascular bed was fixed with 4% formalin (Muto Pure Chemicals), and three sections were prepared randomly in the direction perpendicular to the SFA and SFV. Immunofluorescence staining was performed using primary antibodies against αSMA and CD31 (see below). Four random images were taken at high magnification (×40 objective), and the luminal structures that stained positively for both αSMA and CD31 were counted as blood vessels. The number of blood vessels was measured, and blood vessel density was determined. Vascular smooth muscle area was calculated using NIS-Elements Basic Research (Nikon). All measurements were made in a double-blind manner.

### Generation of hiPSC-derived cardiomyocyte sheets

The 201B7 hiPSC line (Riken)^36,37^ was transfected with the puromycin resistance gene under the control of the mouse alpha-myosin heavy chain promoter and the neomycin resistance gene under the control of the rex-1 promoter, as described previously^36^. The iPSCs were maintained and differentiated into cardiomyocytes using published methods^38^. The cardiomyocytes were purified using a modification of an earlier protocol^36^. Briefly, the cells were cultured in Dulbecco’s modified Eagle medium (DMEM) containing 10% fetal bovine serum (FBS) at 37 °C in a humidified atmosphere containing 5% CO_2_. After 4 days of culture, the cells were treated with 1.5 ng/mL puromycin (Thermo Fisher Scientific) for 24 h. The remaining cells were dissociated using 0.05% trypsin/ethylenediaminetetraacetic acid. The cells were re-seeded on 12-well temperature-responsive dishes (UpCell; CellSeed) at 1.0 × 10^6^ cells/cm^2^ and cultured in DMEM supplemented with 10% FBS for 4 days. The cardiac cells were harvested as a monolayer sheet by lowering the culture temperature to 20 °C. The cell sheet was transferred to another dish and incubated at 37 °C for 30 min to allow it to adhere to the culture surface. Then, a second cell sheet was placed on top of the first sheet, and the medium was aspirated to allow the two cell sheets to adhere to each other. After incubation at 37 °C for 30 min, a third cell sheet was added using the same procedures to create a triple-layered construct.

### Construction of vascularized human cardiac tissue

The vascular bed was induced in male F344/NJcl-rnu/rnu rats weighing 200–380 g (Clea Japan) as described above. On day 21, a three-layer human cardiomyocyte sheet was transplanted onto the vascular bed and encapsulated with an EVOH membrane. The following day, another three-layer sheet was transplanted onto the first three-layer sheet, and the construct was encapsulated with an EVOH membrane. The cell sheets were incubated in vivo for 7 days, following which the rat was euthanized with an overdose of pentobarbital.

### Quantitative evaluation of myocardial tissue thickness and area

The tissue was resected from the rat and fixed with formalin, and three sections were randomly prepared in the direction perpendicular to the SFA and SFV. The thickness of the tissue was measured at three positions (one-quarter, one-half and three-quarters of the way along the long-axis) using NIS-Elements Basic Research. The average of the values at the three locations was used for the analysis. In addition, sections were immunostained for cTnT (see below), and the stained myocardial tissue area and total tissue area were measured using NIS-Elements Basic Research.

### Measurement of the electric potentials and pressure changes generated by human cardiac tissue after its transplantation onto the CIA as a cuff

The rat was anesthetized (inhalation of 3% isoflurane), tracheotomized and mechanically ventilated. A catheter was inserted into the right internal jugular vein, and 1 mL lactated Ringer’s solution was injected every 30 min to maintain hemodynamic stability under electrocardiographic monitoring. To enable the myocardial tissue to be mobilized to the contralateral side of the animal, the abdominal aorta down to the SFA was dissected from the surrounding tissues after all the branches of these arterial vessels had been thermally coagulated or ligated with 6-0 silk. The contralateral CIA was dissected free from the surrounding tissue, and the vascularized myocardial tissue was wrapped around the CIA as a cuff. A catheter with a 1.4 mm microtip pressure transducer (Millar) was inserted from the distal end of the CIA, and a closed circuit was created by clamping the abdominal aorta with bulldog forceps. The electric potentials of the host rat (i.e., electrocardiogram) and the myocardial tissue cuff were recorded using electrodes, and the pressure within the CIA was measured with the catheter transducer. Artefacts due to respiratory movements were suppressed during recording by slightly deepening the anesthesia and temporarily stopping the ventilator (which was restarted immediately after the recording had been made). The electric potentials were amplified by bioelectric amplifiers (UA102, Unique Medical) before acquisition. The electric potentials and intra-arterial pressure were digitized and recorded using an ML870 PowerLab 8/30 data acquisition system and LabChart 7 (ADInstruments).

### Morphological analyses

Tissues were fixed in 4% paraformaldehyde and routinely processed into 7 µm-thick paraffin-embedded sections. Staining with Azan or Sirius red was performed using conventional methods. Immunohistochemistry was carried out in de-paraffinized sections using anti-CD31 rabbit polyclonal antibody (1:10; Life Technologies; used to identify endothelial cells), anti-RECA-1 mouse polyclonal antibody (1:100; Life Technologies; used to identify endothelial cells), anti-cTnT mouse monoclonal antibody (1:100; Thermo Fisher Scientific; used to identify cardiomyocytes), anti-αSMA mouse monoclonal antibody (1:100; Abcam; used to identify smooth muscle cells) or anti-p65 rabbit monoclonal antibody (1:250; Abcam; used to detect NF-κB) overnight at 4 °C. Then, the specimens were treated with Alexa-Fluor-488-conjugated anti-rabbit IgG (1:200; Life Technologies) and Alexa-Fluor-568-conjugated anti-mouse IgG (1:200; Life Technologies) for 40 min at room temperature. Cell nuclei were counterstained by ProLong Gold antifade reagent with DAPI (Life Technologies) for 5 min. Sections were visualized using confocal laser-scanning microscopy (LSM 510 META; Carl Zeiss).

### Video capture

Macroscopic images of pulsatile myocardial tissue grafts were recorded by a motion analyzing microscope (VW5000; Keyence). The videos were edited with Premiere Pro 4.0 (Adobe).

### Data analysis

All data are expressed as the mean ± standard error of the mean (SEM). Student’s *t* test for unpaired samples was used to compare two groups. One-way analysis of variance was used for comparisons of three or more groups; if the *F*-distribution was significant, Fisher’s LSD test was applied to specify the differences between groups. A *p* value of <0.05 was considered significant.

### Reporting summary

Further information on research design is available in the Nature Research Reporting Summary linked to this article.

## Supplementary information

Supplementary Figure 1

movie 1

movie 2

REPORTING SUMMARY

## Data Availability

The data that support the findings of this study are available from the corresponding author on request.
