# Supplementary material for: Bioartificial pulsatile cuffs fabricated from human induced pluripotent stem cell-derived cardiomyocytes using a pre-vascularization technique
Source: NPJ Regen Med. 2022 Mar 31;7:22. doi: 10.1038/s41536-022-00218-7 (PMC8971499; doi:10.1038/s41536-022-00218-7)
Supplement: Supplementary file 1 — Supplementary Figure 1 [file 41536_2022_218_MOESM1_ESM.pdf]

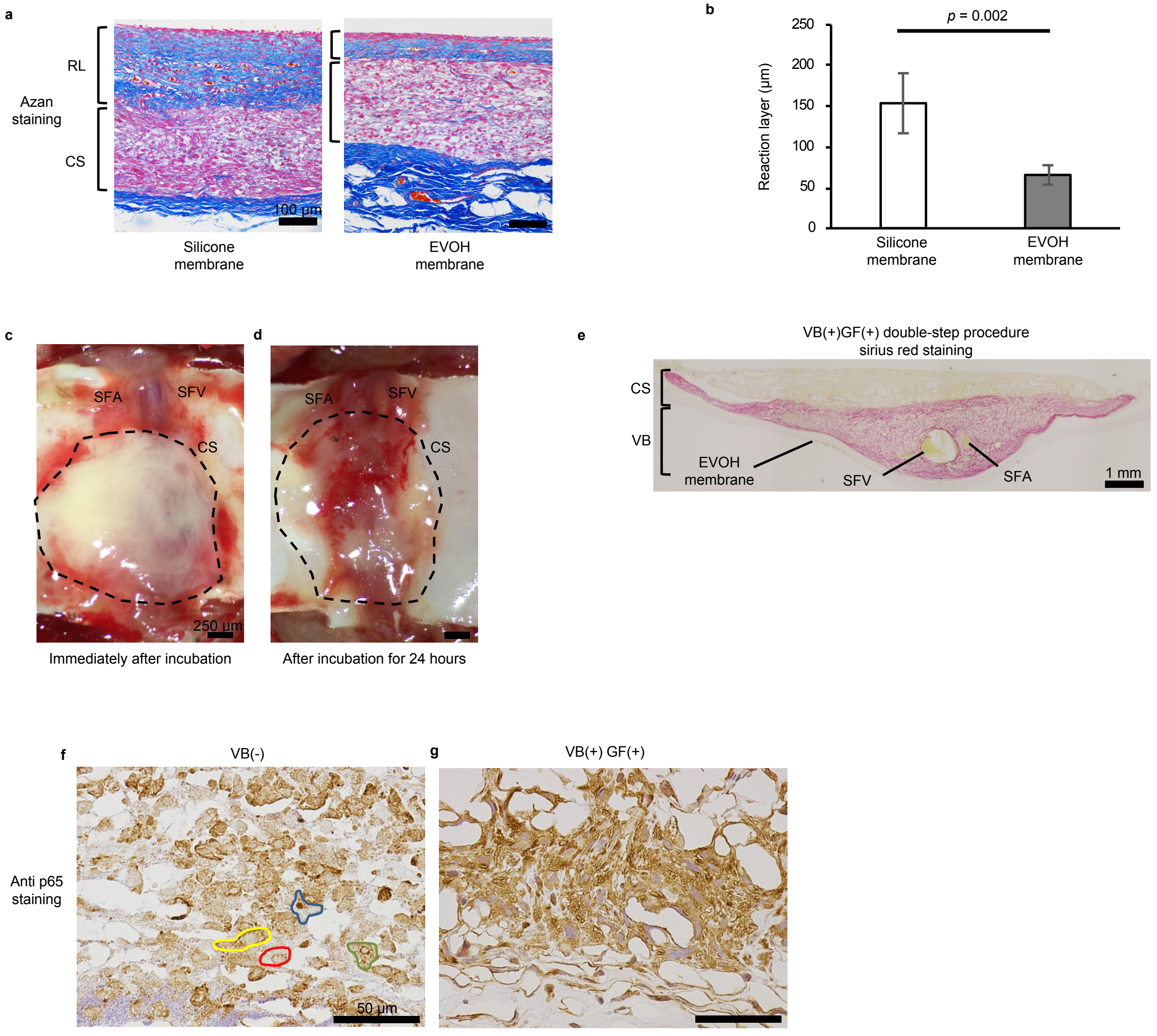

a
RL
Azan
staining
CS
100 µm
Silicone
membrane
EVOH
membrane
b
p = 0.002
250
200
150
100
50
0
Reaction layer (µm)
Silicone
membrane
EVOH
membrane
c
SFA
SFV
CS
250 µm
Immediately after incubation
d
SFA
SFV
CS
After incubation for 24 hours
e
VB(+)GF(+) double-step procedure
sirius red staining
CS
VB
EVOH
membrane
SFV
SFA
1 mm
f
VB(-)
Anti p65
staining
50 µm
g
VB(+) GF(+)

Figure legends

Supplementary Figure. 1.

Evaluation of the vascularized myocardial tissue.

a Staining with Azan was used to identify the reaction layer (RL) formed when triple-layered hiPSC-derived cardiomyocyte sheets (CS) were encapsulated in a silicone membrane or EVOH membrane, transplanted onto the superficial back muscles of rats and cultured *in vivo* for 2 weeks.

b Thickness of the reaction layer (mean ± SEM, n = 5).

c Photograph showing three hiPSC-derived cardiomyocyte sheets (CS) immediately after their transplantation onto the vascular bed.

d Photograph obtained after three hiPSC-derived cardiomyocyte sheets (CS) had been transplanted onto the vascular bed and incubated *in vivo* for 24 hours.

e Six hiPSC-derived cardiomyocyte sheets were transplanted onto the vascular bed (prepared using growth factors) using the two-step method and incubated *in vivo* for 3 days. Staining of the tissue with Sirius red after 3 days did not reveal the presence of fibrosis within the myocardial graft.

f, g Representative images showing sections of myocardial tissue that had been incubated *in vivo* for 3 days (i.e., before fibrosis could develop) and then immunostained for p65. In the VB(-) group (**f**), there was strong staining of the cell membrane (yellow) in some cells,

while other cells showed granular staining in the cytoplasm and around the nucleus (green). Some cells exhibited weak staining of the cytoplasm but strong staining around the nucleus (red) or within the nucleus (blue). The diverse staining patterns likely reflect a series of processes in which p50/p65 dimers are stimulated by tumor necrosis factor receptors and TLRs on the cell membrane to translocate from the cytoplasm to the nucleus, where they regulate transcription. The VB(+) GF(+) group exhibited stronger staining of the cytoplasm and weaker staining of the nucleus.

Supplementary Movie 1.

Flow from the SFA to the SFV via newly formed vessels in the vascular bed.

Fluorescent beads were administered from a catheter in the SFA. The catheter was inserted through the abdominal aorta, and its tip was advanced into the SFA under direct vision until it was just proximal to the vascular bed. A ligature was placed around the SFA to secure the catheter tip and prevent escape of the fluorescent beads. The fluorescent beads administered into the SFA were observed to flow through the newly formed vessels in the gelatin gel and into the SFV (playback speed, 1.5x; objective, x16). This demonstrates the successful creation of a vascular bed that was fed by the SFA and drained by the SFV.

Supplementary Movie 2.

The pumping function of the constructed myocardial tissue.

The myocardial tissue graft was mobilized and wrapped as a cuff around the contralateral common iliac artery. The pressure generated in the common iliac artery by contraction of the myocardial tissue graft was measured using a catheter with a transducer.
