# Supplementary material for: Bioartificial pulsatile cuffs fabricated from human induced pluripotent stem cell-derived cardiomyocytes using a pre-vascularization technique
Source: NPJ Regen Med. 2022 Mar 31;7:22. doi: 10.1038/s41536-022-00218-7 (PMC8971499; doi:10.1038/s41536-022-00218-7)
Supplement: Supplementary file 4 — REPORTING SUMMARY [file 41536_2022_218_MOESM4_ESM.pdf]

nature portfolio

Corresponding author(s): Hidekazu Sekine

Last updated by author(s): Dec 2, 2021

Reporting Summary

Nature Portfolio wishes to improve the reproducibility of the work that we publish. This form provides structure for consistency and transparency in reporting. For further information on Nature Portfolio policies, see our Editorial Policies and the Editorial Policy Checklist.

Statistics

For all statistical analyses, confirm that the following items are present in the figure legend, table legend, main text, or Methods section.

n/a	Confirmed	
☐	☒	The exact sample size (*n*) for each experimental group/condition, given as a discrete number and unit of measurement
☐	☒	A statement on whether measurements were taken from distinct samples or whether the same sample was measured repeatedly
☐	☒	The statistical test(s) used AND whether they are one- or two-sided<br>*Only common tests should be described solely by name; describe more complex techniques in the Methods section.*
☐	☒	A description of all covariates tested
☒	☐	A description of any assumptions or corrections, such as tests of normality and adjustment for multiple comparisons
☐	☒	A full description of the statistical parameters including central tendency (e.g. means) or other basic estimates (e.g. regression coefficient) AND variation (e.g. standard deviation) or associated estimates of uncertainty (e.g. confidence intervals)
☐	☒	For null hypothesis testing, the test statistic (e.g. *F*, *t*, *r*) with confidence intervals, effect sizes, degrees of freedom and *P* value noted<br>*Give P values as exact values whenever suitable.*
☒	☐	For Bayesian analysis, information on the choice of priors and Markov chain Monte Carlo settings
☒	☐	For hierarchical and complex designs, identification of the appropriate level for tests and full reporting of outcomes
☒	☐	Estimates of effect sizes (e.g. Cohen's *d*, Pearson's *r*), indicating how they were calculated

Our web collection on statistics for biologists contains articles on many of the points above.

Software and code

Policy information about availability of computer code

Data collection	The number of vessels, the area and thickness of cardiac tissues were measured using NIS-Elements Basic Research.<br>The electric potentials and intra-arterial pressure were digitized and recorded using an ML870 PowerLab 8/30 data acquisition system and LabChart 7 (ADInstruments).
Data analysis	Numeric date was summarized in Microsoft Excel.

For manuscripts utilizing custom algorithms or software that are central to the research but not yet described in published literature, software must be made available to editors and reviewers. We strongly encourage code deposition in a community repository (e.g. GitHub). See the Nature Portfolio guidelines for submitting code & software for further information.

Data

Policy information about availability of data

All manuscripts must include a data availability statement. This statement should provide the following information, where applicable:

- Accession codes, unique identifiers, or web links for publicly available datasets
- A description of any restrictions on data availability
- For clinical datasets or third party data, please ensure that the statement adheres to our policy

The data that support the findings of this study are available from the corresponding author on request.

Field-specific reporting

Please select the one below that is the best fit for your research. If you are not sure, read the appropriate sections before making your selection.

☒ Life sciences ☐ Behavioural & social sciences ☐ Ecological, evolutionary & environmental sciences

For a reference copy of the document with all sections, see nature.com/documents/nr-reporting-summary-flat.pdf

Life sciences study design

All studies must disclose on these points even when the disclosure is negative.

Sample size	We use the minimum number of animals to reproduce and to fulfill statistical analysis.
Data exclusions	1 out of 8 cases was excluded because of contamination when we made the vascular bed.
Replication	One attempt cannot be reproduced because of contamination, however other attempts were reproduced.
Randomization	All animals were allocated into the different group. We performed the experiments with inguinal regions of rats and alternately left and right.
Blinding	All measurements were made in a double-blind manner.

Reporting for specific materials, systems and methods

We require information from authors about some types of materials, experimental systems and methods used in many studies. Here, indicate whether each material, system or method listed is relevant to your study. If you are not sure if a list item applies to your research, read the appropriate section before selecting a response.

Materials & experimental systems

n/a	Involved in the study	
☐	☒	Antibodies
☐	☒	Eukaryotic cell lines
☒	☐	Palaeontology and archaeology
☐	☒	Animals and other organisms
☒	☐	Human research participants
☒	☐	Clinical data
☒	☐	Dual use research of concern

Methods

n/a	Involved in the study	
☒	☐	ChIP-seq
☒	☐	Flow cytometry
☒	☐	MRI-based neuroimaging

Antibodies

Antibodies used

Tomato lectin (DyLight 594-conjugated Lycopersion esculentum Lectin; Funakoshi)
fluorescent beads (FluoSpheresTM sulfate, 4.0 µm, yellow-green; Life Technologies)
anti-CD31 rabbit polyclonal antibody (1:10; Life Technologies; used to identify endothelial cells)
anti-RECA-1 mouse polyclonal antibody (1:100; Life Technologies; used to identify endothelial cells)
anti-cTnT mouse monoclonal antibody (1:100; Thermo Fisher Scientific; used to identify cardiomyocytes)
anti-αSMA mouse monoclonal antibody (1:100; Abcam; used to identify smooth muscle cells)
anti-p65 rabbit monoclonal antibody (1:250; Abcam; used to detect NF-κB),

Alexa-Fluor-488-conjugated anti-rabbit IgG (1:200; Life Technologies)
Alexa-Fluor-568-conjugated anti-mouse IgG (1:200; Life Technologies)
DAPI (Life Technologies)

Validation

(Abcam)
Antibody validation must be application-specific to be effective and information on which applications an antibody has been validated in can be found in the Tested Applications section on any antibody datasheet.

(Life Technologies, Thermo Fisher Scientific)
The Validation and Verification Atlas Resource organizes information on forensic DNA analysis methods and provides direct access to Life Technologies' developmental validation studies, reference materials, quality assurance guidelines, user bulletins, user manuals, and other supporting documentation.

Certificate of analysis of Tomato lectin can be available on web (https://fnkprddata.blob.core.windows.net/domestic/data/datasheet/VEC/L-1170.pdf).

Eukaryotic cell lines

Policy information about cell lines

Cell line source(s)	The 201B7 hiPSC line (Riken) was used.
Authentication	We purchase from Riken Bioresource
Mycoplasma contamination	The cell lines were not tested for mycoplasma conatamination.
Commonly misidentified lines (See ICLAC register)	none

Animals and other organisms

Policy information about studies involving animals; ARRIVE guidelines recommended for reporting animal research

Laboratory animals	Male Jcl:SD rats weighing 270–470 g (Clea Japan)<br>male F344/NJcl-rnu/rnu rats weighing 200–380 g (Clea Japan)
Wild animals	The study did not involve wild animals.
Field-collected samples	The study did not involve samples collected from the fields.
Ethics oversight	All animal experiments were performed in accordance with the "Guidelines of Tokyo Women's Medical University on Animal Use" and were approved by the Ethics Committee for Animal Experimentation of Tokyo Women's Medical University.

Note that full information on the approval of the study protocol must also be provided in the manuscript.
